# A Reflection

**Published:** 1921-04-09

**Authors:** 


### THE PRACTICE OF BIRTH-CONTROL.

### A Reflection.

In North London a new kind of clinic—new as far as Britain is concerned—has just been started with the object of teaching the proletariat how to prevent conception. This enterprise has been formed by Mr. H. V. Roe and his wife (better known as Dr. Marie Stopes), were kind enough to invite the Editor of The Hospital to the opening of the clinic; unfortunately the date and time precluded the possibility of accepting the invitation, and the representative who was asked to visit the clinic a few days later unfortunately arrived at a time when it was closed. It is, therefore, purely from theoretical considerations, and by the pamphlet which Mr. and Mrs. Roe have issued, that the following reflections are prompted.

### The New Clinic.

The clinic has been started for the express object of teaching people how to control conception—that is, how to prevent it. "Only Motherhood which is in the control of the mother can now truly advance our race," says the pamphlet. A great deal that is perfectly true is brought forward in support of this teaching—such as that too rapidly recurring pregnancies exhaust a mother and tend towards a high infantile death-rate. Let it be admitted at once that a lessened birth-rate in our slums would bring with it certain advantages, some of them far from negligible.

It is necessary, however, to look a little further ahead, if that be possible. Amongst the educated, and also by now the semi-educated classes, the knowledge which this clinic seeks to disseminate is widespread—practically universal. The result is that a family of three is now regarded as exceedingly large among the professional and business classes. Any practitioner can certify the truth of this; and it follows that, when the unmarried and the sterile married are deducted, the middle classes of Britain are actually failing to reproduce themselves. This is no mere hypothetical but unlikely contingency—it is an actual fact. If this, then, is the result of the knowledge among the most intelligent types of how to prevent conception, what are the probable results of disseminating similar knowledge among the less intelligent? Surely that race suicide will spread to them too.

To many social reformers this possibility presents no terrors—they say we have too large a population as it is, and that lower quantity with greater quality is what we want. If Britain were the only nation in the world this argument might hold water. But the consequences of race suicide stare us in the face. France has tried it for two generations, and has paid dearly for it. Germany is thirsting for revenge, and clearly has no intention of making restitution for the wrongs she did in 1914-18 unless under direct compulsion. Race suicide here means the triumph in thirty or forty years of either the Teuton or the Slav—unless they catch the contagion too, as is quite probable, in which case it means the triumph of the yellow races, and, if they catch it, of the negro. And racial triumph hereafter is going to be no affair of mild displacement and absorption, as it has been in the past. If the whites decline in numbers they may be able for a while to retain their place in the sun by reason of superior inventiveness. But a time may come when even the chemist and the engineer can no longer make up for hopeless inferiority of numbers, and the Indo-European races may be wiped off the face of the earth because their women would not conceive children.

### Waste Spaces of the Earth.

This is the picture that we would commend in all seriousness to the attention of "birth-control" enthusiasts. To call it fantastic or far-fetched is no answer. The plain fact is that when civilised women once learn how to prevent conception they will not consent to conceive sufficiently often to keep their race going, let alone to colonise the waste spaces of the earth.

Marie Stopes was "converted" to a belief in the advantages of birth-control (according to her statement) by an incident in a hospital out-patient room. A woman student saw a mother with a wilted baby which was, in the student's eyes, "obviously" suffering from syphilis. The doctor in charge told the mother in answer to her direct question that there was nothing wrong with her husband. Clearly the doctor did not believe that the case was one of syphilis; or else he lied. Dr. Stopes would not care to accept the lightning diagnosis of a medical student in preference to the considered opinion of a (presumably) experienced hospital teacher about her own kith and kin, we take it: she would not accept the verdict of one of her own students in opposition to her own judgment about a point in palæo-botany; why should she base a complete change in her opinions about birth-control on evidence that nobody would swing a cat upon? If the baby in the case she quotes was syphilitic it was the doctor's business to cure it and its parents too, and not to tell lies; but the evidence that he failed in his duty in either respect is really non-existent, and if he did do so we cannot for the life of us see that it helps Dr. Stopes' cause. We are not saying that Mr. Roe and his wife are doing a thing which is wholly anti-social: merely that some of the arguments they rely on seem to us exceedingly weak, and that there are arguments to the contrary which their pamphlet shows no signs that they have considered.

